# Preparation of Epoxy Resin with Disulfide-Containing Curing Agent and Its Application in Self-Healing Coating

**DOI:** 10.3390/ma16124440

**Published:** 2023-06-16

**Authors:** Baolei Wang, Zewei Li, Xinru Liu, Lulu Li, Jianxiang Yu, Shuang Li, Gaiping Guo, Dahai Gao, Yuhua Dai

**Affiliations:** Beijing Key Laboratory of Special Elastomeric Composite Materials, Beijing Institute of Petrochemical Technology, College of New Materials and Chemical Engineering, Beijing 102617, China

**Keywords:** self-healing polymer, epoxy resin, dynamic covalent bond, healing efficiency

## Abstract

Intrinsic self-healing polymers via dynamic covalent bonds have been attracting extensive attention because of their repeatable self-healing property. Herein, a novel self-healing epoxy resin was synthesized with disulfide-containing curing agent via the condensation of dimethyl 3,3′-dithiodipropionate (DTPA) and polyether amine (PEA). Therefore, in the structure of cured resin, flexible molecular chains and disulfide bonds were imported into the cross-linked polymer networks for triggering self-healing performance. The self-healing reaction of cracked samples was realized under a mild condition (60 °C for 6 h). The distribution of flexible polymer segments, disulfide bonds and hydrogen bonds in cross-linked networks plays a great role in the self-healing process of prepared resins. The molar ratio of PEA and DTPA strongly affects the mechanical performance and self-healing property. Especially when that molar ratio of PEA to DTPA is 2, the cured self-healing resin sample showed great ultimate elongation (795%) and excellent healing efficiency (98%). The products can be used as an organic coating, in which the crack could self-repair during a limited time. The corrosion resistance of a typical cure coating sample has been testified by an immersion experiment and electrochemistry impedance spectrum (EIS). This work provided a simple and low-cost route to prepare a self-healing coating for prolonging the service life of conventional epoxy coatings.

## 1. Introduction

Inspired by natural biomaterials such as human skin, bones and the stratum corneum of insects [1], self-healing polymers based on reversible dynamic covalent bonding and supramolecular interactions have drawn great attention in the last decade [2]. Recently, various self-healing materials have been widely applied in crucial fields, such as field effect transistors [3], solar cells [4], electrochemical sensors [5], electric cells [6], supercapacitors [7,8], coatings [9,10] and other fields. Dynamic covalent bonds and supramolecular links allow the repeated healing of polymer-based materials for prolonging the life time of those materials. The type of material is noteworthy because self-healing shows great importance in ecology, where raw materials and energy could be saved during their long-term use. Researchers are able to constitute a polymer framework containing reversible links based on numerous polymer structures, especially on polyurethane, polyester, epoxy and light-initiated polymers.

Thermosetting epoxy resins are widely used in anti-corrosion coatings, high performance adhesives, insulation materials and electrical packaging materials, owing to their excellent mechanical strength, thermal stability and chemical resistance [11,12]. However, these products will inevitably suffer minor damage during use. These damages, if not detected in time, can easily generate macroscopic cracks, which greatly reduce the mechanical strength of the epoxy resin, leading to serious safety accidents. Therefore, it is of vital importance to provide self-healing properties to them for extending the service life of the material. The self-healing occurs spontaneously by reversible bond breaking and recombination in response to an external stimulus. In fact, such reversible dynamic interactions have been used extensively in organic synthesis, materials science and in biomedical applications [13,14], which frequently use polymeric materials due to their advanced properties, such as ductility, self-healing, shape memory, adaptability, stress relaxation and responsiveness [15].

Many researchers have aimed to integrate reversible covalent bonds into the framework of epoxy resin. Typical reversible covalent bonds include imine bonds [16], Diels-Alder reactions [17], borate ester bonds [18], disulfide bonds [19], diselenium bonds [20] and urea bonds [21], among others. Strachota et al. [22] designed a dual network self-healing thermosetting material based on the Diels-Alder (DA) reversible network and an epoxy network, showing a healing efficiency close to 100%. A bio-based curing agent with reversible imino-containing bonds was synthesized by Rashid et al. [23]. The imine groups among a three-dimensional network of epoxy resin show a considerable low activation energy, which provide excellent crack repair efficiency (up to 80%). Zeng et al. [24] synthesized a novel epoxy network comprising borate ester bonds. With the help of the borate ester exchange reaction, the prepared polymers show good performance in self-healing and shape memory to ensure their accessibility in welding and recycling.

Despite the ability of self-healing epoxy resins constructed based on various dynamic covalent bonds to provide high repair efficiency and reproducibility, problems such as ester hydrolysis, catalyst leaching and harsh repair conditions restrict the wide application of self-healing epoxy resins. Compared with most types of dynamic covalent bonds, the disulfide bond (S-S) shows chemical stability and low bond energies, which are prone to recombine in the cracks of a polymer matrix at relative lower temperature [25,26]. Disulfide bonds can be imported into the polymer networks via a binder resin or curing agent. Li [27] et al. used amine curing agents containing disulfide bonds to crosslink the hydrogenated bisphenol A epoxy resin and polypropylene glycol diglycidyl ether as hard and soft segments, respectively, to obtain the crosslinked network. By introducing the soft segment of polypropylene glycol diglycidyl ether, the mobility of the molecular chains in the crosslinked network was improved, which contributed to a self-healing efficiency of 71.5%. Wang et al. [28] prepared carboxylic acid-extended epoxy resins which incorporated dynamic *β*-hydroxy esters via condensation between bisphenol A epoxy resin and sulphite (SA) using an aromatic disulfide as curing agent. The products exhibited excellent and sensitive self-healing properties with a self-healing time of only 10 min and a self-healing rate up to 80%. Through the oxidation of multifunction thiols by iodine (III), Ortiz et al. [29] prepared thioether disulfide oligomers, which allow the repair of specimens at a temperature less than 80 °C. Various molecular designs can further optimize the self-healing performance and condition; however, the simple routes to import disulfide bonds into epoxy backbones are still challengeable.

In this study, we conducted the condensation of polyetheramine (PEA) D400 and dimethyl 3,3-thiodipropionate (DTPA) as soft segments. The products were applied as disulfide-containing curing agents. The proportion of the flexible segment in the cross-linked network was controlled by adjusting the content of PEA in order to prepare a novel self-healing epoxy resin. The products are expected to become candidates of self-healing anti-corrosion coatings.

## 2. Materials and Methods

### 2.1. Materials

Dimethyl 3,3′-dithiodipropionate (DTPA,96%) was purchased from Shanghai Aladdin Biochemical Technology Ltd. (Shanghhai, China). Diamine-terminated poly (propylene glycol) bis (2-aminopropyl ether) (D-400) with an average molecular weight of 400 and an amine value of 0.450 mol/100 g was purchased from Shanghai Aladdin Biochemical Technology Ltd. Polyurethane-modified epoxy resin (SL3412; epoxy value 0.43 mol/100 g) was supplied by Zhuzhou Shilin Polymer Ltd. (Zhuzhou, China). 2,4,6-Tris(dimethylaminomethyl)phenol (CP) was provided by Sinopharm Group Chemical Reagent Ltd. (Shanghai, China). Anhydrous ethanol (AR; ≥99.7%) was bought at Shanghai Titan Technology Ltd. (Shanghai, China). Hydrochloric acid (AR; 36–38 wt%) was supplied by Beijing Reagent Ltd. (Beijing, China). The raw materials and reagents were used without further purification.

### 2.2. Preparation of Disulfide-Containing Curing Agent (PD)

In a typical procedure, polyetheramine D400 (14.4 g, 36 mmol) was added to a 50 mL three-necked flask equipped with a condenser tube and magnetic stirrer under an argon atmosphere. Dimethyl 3,3-dithiodipropionate (3.91 mL, 20 mmol) was added dropwise from a pressure-equalizing dropping funnel under an ice-water bath. The reaction mixture was transferred to an oil bath for 6 h at 100 °C. All the products were collected and were labeled as PD-1 to -5 based on the molar ratio of PEA and DTPA. The formulations are detailed in Table 1.

### 2.3. Curing of Self-Healing Epoxy Resin

**Scheme 1:**
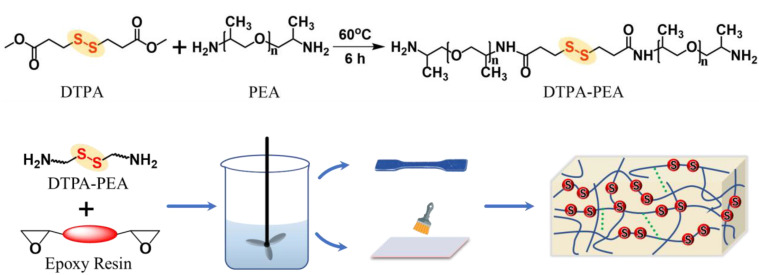
The preparation of a disulfide-containing curing agent and self-healing epoxy resin. The condensation products of DTPA and PEA are used as curing agents of epoxy resin. The structure of cured resin contains disulfide bonds and hydrogen bonds.

Epoxy resin SL3412 was mixed with prepared PD in a proportion calculated by Equation (1):

(1) $$m=\frac{M_{n}}{n}\times E_{v}$$

where the weight of curing agent *m* is corresponding to 100 g of EP resin, the molar weight of curing agent *M_n_* is theoretical molecular weight of curing agent (according to structural formula in Scheme 1), *n* is the number of active hydrogen atoms in curing agent molecules and *E_v_* is the epoxy value (the mole number per 100 g of resin). 2,4,6-tris(dimethylaminomethyl)phenol (1–2 wt% of cured epoxy resin) was added dropwise to accelerate the curing reaction. The mixture was then transferred to a vacuum blender (SIE MIX60, Guangzhou Sienox Science and Technology Ltd., Guangzhou, China) and was defoamed and dispersed at 3000 rpm for 5 min. The products were immediately poured into a Teflon model and painted onto glass and metal substrates. All the shaped and coated samples were cured for 24 h at 60 °C.

### 2.4. Characterizations

The chemical structure of prepared products was characterized by Fourier transform infrared spectroscopy (FTIR; Nicolet IS5, 4000–400 cm^−1^), ^1^H-NMR (Germany-Bruker-Avance III HD 400 M, Bruker 400 MHz) and Raman spectroscopy (Horiba scientific LabRAM HR evolution RAM, 532 nm, 400–1800 cm^−1^). ^1^H-NMR was conducted at 25 °C with CDCl_3_ as the solvent and tetramethylsilane as an internal reference. Thermogravimetric analysis (TGA) and differential scanning calorimetry (DSC) of the samples were performed using a Setline thermogravimetric analyzer (TGA, 25–800 °C, 10 °C/min, N_2_; DSC, −30–90 °C, 5 °C/min). Dynamic thermal mechanical analyses (DMTA) of PD-1 to 5 were conducted on a Mettler-Toledo DMA 1 instrument (−30–100 °C, 5 °C/min, tensile mode). The storage and loss modulus can be recorded by DMTA data, and tan *δ* is the ratio of loss modulus to storage modulus.

The mechanical properties were tested on the GP-6220 tensile testing machine at room temperature (25 °C) with a tensile speed of 100 mm/min. All the test procedures were performed according to the standard GB/T 1040.2-2022 [30]. The dumbbell-shaped samples cured in Teflon mold (40 × 5 × 2 mm) were used for the tensile test. Each test was repeated three times, and the measured values of tensile strength and elongation at break were recorded. To evaluate the self-healing properties, the specimens were cut at the central part with a surgical blade, and the cut surfaces were joined by clamps at 60 °C for 6 h to self-repair. The self-repair efficiency was calculated by the quotient of tensile strength after and before self-healing, as shown in Equation (2):

(2) $$\eta =\frac{\sigma_{1}}{\sigma_{2}}\times 100\%$$

where *η* represents the self-repair efficiency, *σ*_1_ represents the tensile strength of cured resin after repair, and *σ*_2_ represents the tensile strength of the original cured resin.

The coated samples were fabricated on glass slides and metal substrates. An SXP-X5 polarizing optical microscope was used to record the self-healing performance of prepared samples on glass slides. All the tests on coating resistance were conducted on the metal substrates.

Coating resistance test: coating samples were immersed in specified liquid medium including deionized water, EtOH, THF, H_2_SO_4_, NaOH and NaCl at a concentration of 1 mol·L^−1^ according to GB/T 30648.1-2014 [31] for 72 h, and then the surface morphology was observed. Coating mechanical properties: the pencil hardness test was performed according to GB/T 6739-1996 [32], and the adhesion test was carried out by the method in GB/T 9286-1998 [33]. Such tests mentioned above were performed on tinplate substrates. Corrosion resistance was determined by electrochemical impedance spectroscopy (EIS) using a Wuhan Corrtest CS350 electrochemical workstation, and the coating substrate (carbon steel plates) was used as the working electrode, platinum plate as counter electrode and the Ag/AgCl electrode as the reference electrode. Herein, 1 cm^2^ of coating area was exposed to the electrolyte solution (3.5 wt% NaCl aqueous solution) in the test device.

## 3. Results and Discussion

### 3.1. Chemical Structure of the Curing Agent

**Figure 1:**
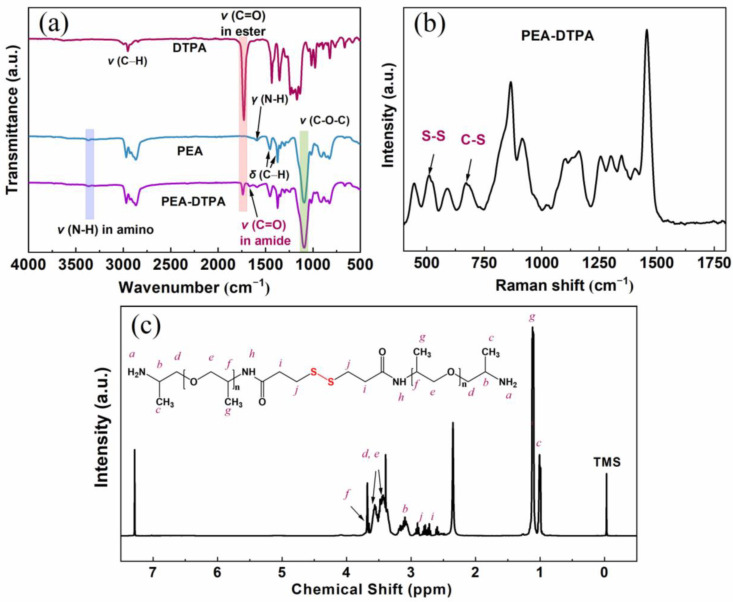
The structural characterization of a prepared curing agent (PD-3). The molar ratio of PEA and DTPA is 2. (**a**) FTIR, with the spectra of DTPA and PEA as control samples, (**b**) Raman spectrum, (**c**) ^1^H-NMR.

The preparation of this work is demonstrated in Scheme 1. Herein, disulfide bonds were imported into the curing agent molecules by the formation of amido (CO-NH). In order to verify the condensation between DTPA and PEA and the existence of -S-S- in the prepared curing agent, FTIR analyses of raw materials and products were performed. Figure 1a displays the FTIR spectra of PD, PEA and DTPA. The spectrum of DTPA shows bands at 1137 cm^−1^, 1729 cm^−1^ and 2953 cm^−1^, corresponding to the vibration of C-O-C, C=O and C-H, respectively. The stretching vibration of C-O-C at 1092 cm^−1^, C-N at 1229 cm^−1^ and the anti-symmetric and symmetric vibrations of C-H at 2868 cm^−1^ and 2969 cm^−1^, respectively, can also be seen in the spectrum of PEA. The bands in the spectrum of the curing agent (PEA-DTPA) contain the same signals as the spectra of DTPA and PEA, but also show evidence of an amino group at 1667 cm^−1^, thus confirming the successful reaction of dimethyl DTPA and PEA.

From the Raman spectrum of PD in Figure 1b, it can be observed that the characteristic absorption band of S-S and C-S bonds appear at 510 cm^−1^ and 650 cm^−1^, respectively, thus indicating that the disulfide bonds have been successfully imported into the molecular backbones of curing agent PD. The ^1^H-NMR spectrum of PEA-DTPA is illustrated in Figure 1c, in which the attributions of H atoms in the curing agent molecule were labeled. The peak at 2.84 ppm could be attributed to the H atom in -S-CH_2_, indicating that the structure of PEA-DTPA contains disulfide bonds. It should be clarified that the molecular structure in Figure 1c is only one form of curing agent molecule. The wide, overlapped peaks in Figure 1c suggest that the products are a mixture of oligomers containing various numbers of PEA and DTPA units, which are caused by the randomness of the condensation reaction. However, we could verify the combination of PEA and DTPA according to the results of all structural characterizations.

### 3.2. Thermal Properties of Self-Healing Epoxy Resin

**Figure 2:**
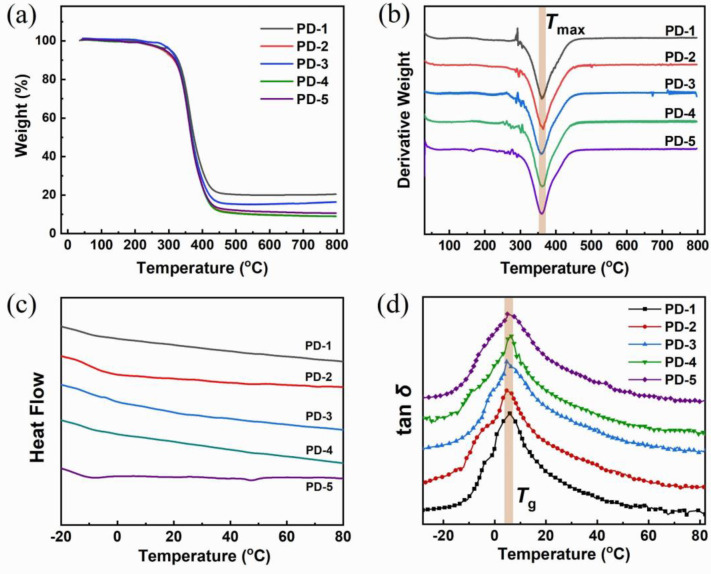
Plots of thermal properties of cured resin containing disulfide-containing curing agent: TGA (**a**), DTG (**b**), DSC (**c**) and DMTA (**d**) The curves were labelled by the curing agent used (PD-1 to 5). *T*_max_ is the temperature at maximum weight loss and *T*_g_ is glass transition temperature.

The cured splines using a series of curing agents (PD-1 to -5) were tested by TGA, DSC and DMTA. In Figure 2a, all of the TGA curves indicate that the initial decomposition happens at 200–250 °C, revealing that the products show sufficient thermal stability below 200 °C. Figure 2b displays the curves of differential thermogravimetric analysis, suggesting that only one main decomposition process occurs during the temperature increase, and the temperature of maximum weight loss is around 360 °C. By comparing the weight loss of different resin samples, PD-3 provided the best thermal stability for the cured resin, which could be attributed to the appropriate degree of crosslink formation in the resin preparation. In that case, PEA just condensed with an equivalent amount of DTPA, yielding amino-ended molecules for curing epoxy resin. The curing agent was incorporated into the backbones of the curing product, which provides a gigantic crosslinking scale, leading to the best thermal stability.

Figure 2c depicts the DSC curves of disulfide-containing resin samples. No obvious signals were observed from room temperature to 80 °C because no glass transitions and chemical reactions occurred in that temperature region, which suggests that the products show an elastomeric state above room temperature. In that case, the molecular segments could slide without overcoming a great energy barrier, which is helpful to repair the breakage. That allows the self-healing reaction to occur at a low temperature. However, DSC curves have not provided sufficient information on glass transition. In fact, we observed glass transition at the region of 4–7 °C, which could be recognized at the peaks of tan *δ* according to DMTA plots, as Figure 2d displays. It was found that the values of glass transition temperature (*T*_g_) are close to each other, but show a slight difference. The *T*_g_ of cured resin using PD-3 reaches the lowest value (4.6 °C), reflecting the excellent flexibility of backbones in that case.

### 3.3. Mechanical and Self-Healing Properties of Cured Disulfide Epoxy Resin

**Figure 3:**
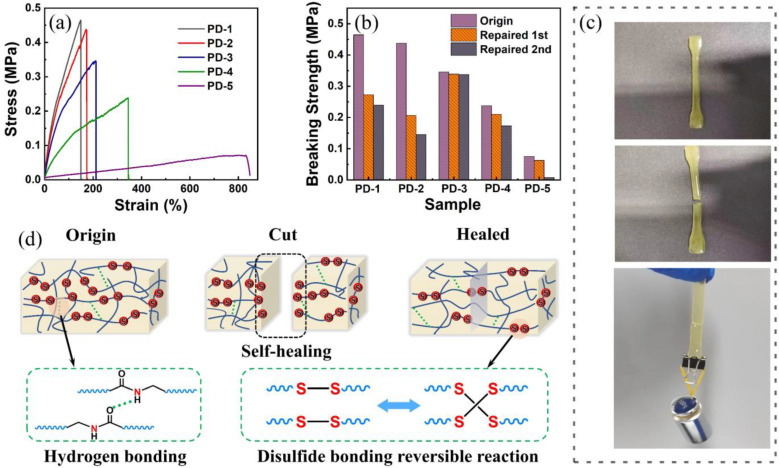
Self-healing performance of prepared disulfide-containing epoxy resin: (**a**) stress-strain curves of prepared samples. (**b**) self-healing efficiency of prepared samples. (**c**) photographs of self-healing process and the weight-bearing test when the healed sample was subjected to a 100 g weight. (**d**) the mechanism of self-healing epoxy resin containing disulfide bonds and hydrogen bonds.

The thermal self-healing properties of prepared epoxy samples were primarily investigated by the tensile-test. Herein, the amounts of curing agent mixed in prepared samples are precisely calculated according to Equation (1). Figure 3a shows the stress-strain curves of original samples, revealing the influence of curing agent composition. Among the molecular structure of curing agents PD-1 to -5, the PEA segment offers flexibility to the resin backbone, while DTPA provides the possibility of bonds breaking and recombination. It was clearly shown that the tensile strength reduces from 0.46 MPa to 0.08 MPa monotonously with the increasing molar ratio of PEA and DTPA, while showing a tendency to rapidly increase on elongation at break (795% for the cured sample using PD-3). In fact, PEA is an amino-ended polyol which has two functional groups. When the molar ratio of PEA and DTPA surpasses 2, more flexible segments are imported into the polymer backbones, which could improve the material flexibility and reduce its modulus, leading to the enhancement of molecule mobility. It could be speculated that flexible segments provide more possibility to recombine the fractured bonds, allowing good self-healing properties.

Indeed, suitable flexibility was found to be an important factor to realize acceptable self-healing performance. Figure 3b compared the tensile strength values of original and repaired samples, which reveals that PD-3 provides the highest self-healing efficiency (98%) at its first self-repairing. The self-healing efficiency also displays a significant trend with the increasing ratio of PEA and DTPA. Figure 3c indicated that the typical resin sample was able to bear a weight of 100 g, which is strong evidence of disulfide recombination. The self-healing mechanism of prepared self-healing samples is demonstrated in Figure 3d, which suggests that the dynamic covalent bonds (disulfide) and hydrogen bonds account for the self-healing mechanism. However, both could not determine the extent of self-healing. Furthermore, the breaking and recombination of disulfide bonds required the movement of polymer chains. Thus, improving flexibility contributes to the enhanced probability of bond recombination. However, when that ratio is larger than 2, no sufficient disulfide bonds are imported into resin backbones, leading to decreased chances of molecules breaking and recombining.

### 3.4. Properties and Performance of Cured Self-Healing Coating

**Figure 4:**
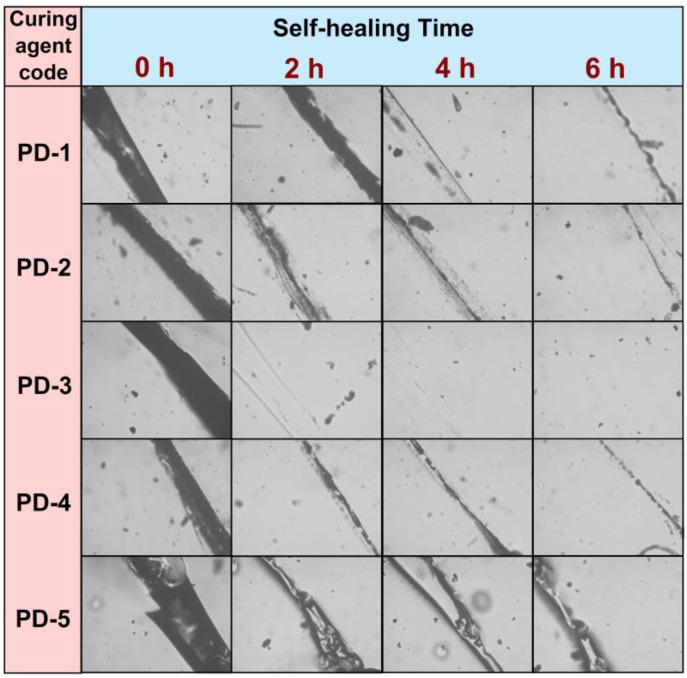
Microscopic photographs of self-healing coated samples prepared by disulfide-containing curing agents with a 10× magnification lens. The coatings were painted onto glass slides, and the formed dry films were cut by a surgical blade. The self-healing of samples was conducted at 60 °C.

For testing the performance of obtained disulfide-containing epoxy resin as an organic coating, coatings on glass slides were prepared. The coated samples were prepared on glass slides to further verify the self-healing properties, which could be observed in an optical microscope. Initially, the coatings were cut by surgical blade to form a scratch with a dimension of 200 μm. After self-healing at 60 °C for a given time (0 h, 2 h, 4 h and 6 h), the photographs of coating morphology were recorded in Figure 4. Generally, the cracks can be repaired to some extent at 60 °C. It was clearly shown that the curing agent PD-3 permits the coating film to give the best self-healing property, which also corresponds to the results of the tensile test. The scratch of this sample was recombined in 2 h, leaving a flat, transparent paint film. The photographs show a similar tendency with the results of self-healing efficiency. In addition, the self-healing behavior of samples cured by PD-1 and PD-5 show considerably wide cracks after healing for 6 h, while the cracks on the coatings cured by PD-2 and PD-4 seem to be more indistinct in their edges. That further suggests that the dynamic bonds and backbone flexibility become balanced when a suitable molar ratio of PEA and DPTA is applied. Other coated samples have shown similar performance during thermal repair; that is, the fracture parts have been recovered to some extent. However, the velocity of crack recombination cannot surpass the product cured by PD-3.

It could be similarly explained by the synergetic effect of repaired groups (disulfide bonds and hydrogen bonds) and flexible segments, as Figure 3d shows. Luo et al. [34] claim that the first step of self-healing could be considered cohesive healing movement, which can be promoted by flexible and mobile chains. Once the cracks were close enough to each other, the second step, that is, disulfide bond exchange, could be activated. Hydrogen bonds were formed between -NH- groups and oxygen atoms in carbonyl groups, which are also dynamic interactions and assist in the realization of the self-healing mechanism. It was found that only a suitable molar ratio of DTPA and PEA contributes to satisfactory self-healing performance. This ratio effectively promotes movement of molecular segments and recombination of bonds after crack formation. For that reason, both factors (dynamic bonds and flexibility of molecular chains) reach a good compromise, which play a key role in the design and fabrication of self-healing polymer materials.

**Figure 5:**
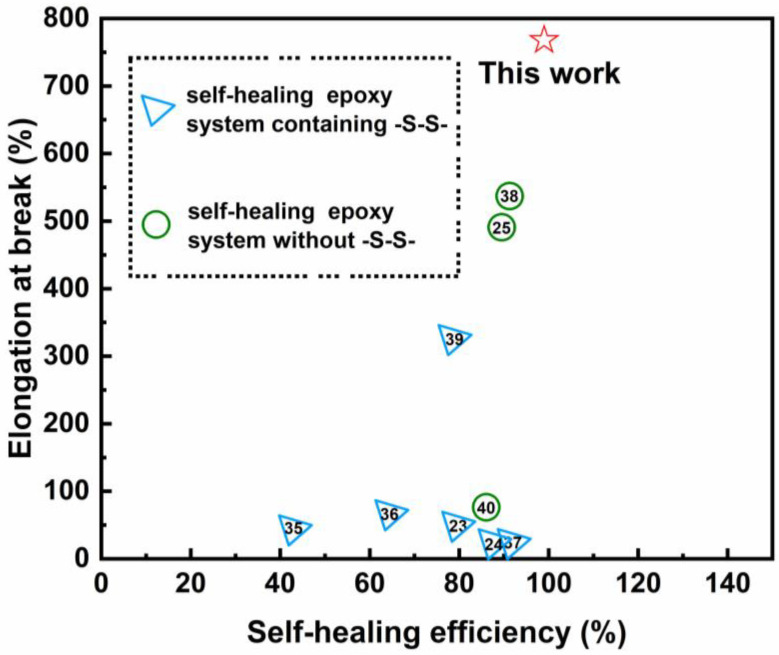
The comparison of self-healing efficiency and elongation at break in recent works [23,24,25,35,36,37,38,39,40] on self-healing epoxy resin.

After we investigated the self-healing epoxy resin, we compared the work in this paper with that in other papers, as shown in Figure 5. We found that the self-healing epoxy resin prepared in this paper has the outstanding advantages of great elongation at break (795%) and extremely high self-healing efficiency (98%). Such excellent mechanical performance and self-healing properties are rare in similar published papers. The values of self-healing efficiency and elongation at break in other recent works on self-healing epoxy resin are compared in Figure 5.

As shown in Table 2, the mechanical properties of coated samples are entirely consistent with the results obtained by the tensile test. Owing to the good flexibility and interface hydrogen bonds, coating films show excellent adhesion to the metal plates. The results of pencil hardness have a similar tendency with the values of tensile strength, which is obviously attributed to the change in the proportion of soft and hard segments in curing agent molecules. We also investigated the coating morphology after immersing in corrosive liquid medium, including common aqueous and organic solutions. The results indicated that the resin backbones cannot be broken by corrosive medium such as NaCl, and NaOH during 3 days, but the structure of cured resin is easily broken when the liquid medium is H_2_SO_4_, EtOH and THF. Therefore, the prepared coating samples show chemical stability in some familiar corrosion environments.

**Figure 6:**
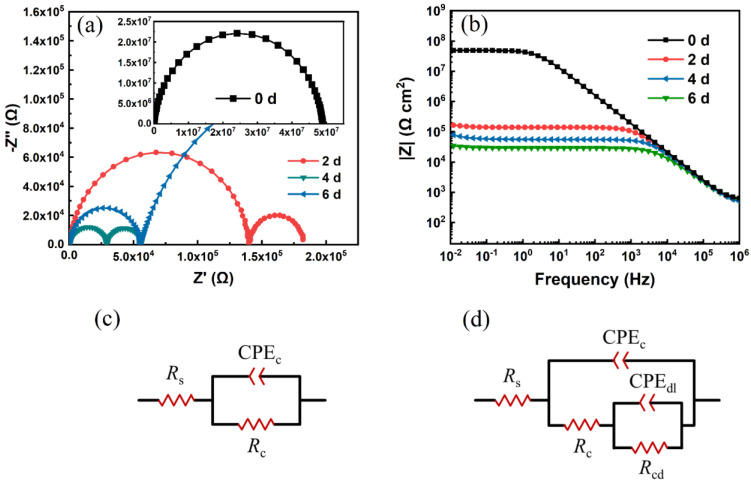
Electrochemical impedance spectra (EIS) of prepared epoxy coatings cured by PD-3 at different immersion times in 3.5 wt% NaCl solution: (**a**) Bode plots, (**b**) Nyquist plots. The thickness of coatings is 30 ± 5 μm. The equivalent circuit models to fit the EIS spectra: (**c**) after 0 days in 3.5 wt% NaCl solution, (**d**) after 2, 4 and 6 days of immersion in 3.5 wt% NaCl solution. *R*_s_, *R*_c_, *R*_cd_ represent solution resistance, coating resistance and charge transfer resistance, respectively. CPE_c_ and CPE_dl_ are the symbols of constant phase elements of coating and double layer.

In Figure 6, the EIS plots (Nyquist and Bode plots) and corresponding equivalent circuit models were demonstrated to display the anti-corrosion performance of prepared self-healing coatings immersed in 3.5 wt% NaCl solution. The Nyquist plots (Figure 6a) imply two time constants, indicating the solution reached the interface of the substrate without destroying the coating film. The radius of the first semi-circle became smaller after immersion over time, which suggests a change of coating resistance. It was well known that the impedance modulus at low frequency (${\left|Z\right|}_{0.01\;\mathrm{Hz}}$) in Bode plots reveals the anti-corrosion property during immersion in corrosive solution. As the Bode plots show (Figure 6b), the ${\left|Z\right|}_{0.01\;\mathrm{Hz}}$ of prepared coatings reduce from 5.2 × 10^7^ to 3.5 × 10^4^ Ω·cm^2^ during 144 h of treatment. The impedance data indicates that the polymer backbones can provide a sufficient barrier effect at initial immersion. Moreover, the rapid decrease in the coating barrier effect could be attributed to the flexibility of polymer backbones, because the motivation of molecule segments permits water penetrating inside the coating. In addition, the polyether part in curing agent molecules show hydrophilic properties, which accelerates the penetration of aqueous medium.

In this work, the compromise of self-healing and molecular flexibility is frequently mentioned to explain the results, including mechanical properties, self-healing rate and efficiency. It should be noted that that the mechanical strength should be enhanced to satisfy the requirements of application, especially as anti-corrosion coatings. However, for the other side, the molecule flexibility facilitates the self-healing performance. That appears to be a contradiction. Therefore, the equilibrium of dynamic bonds and molecular flexibility should be modified by other structural design approaches, such as changing the length of flexible segments, adjusting the number of dynamic groups, and linking with the functional groups by supramolecular effects. We have been taking these approaches in recent works.

## 4. Conclusions

In this study, we have prepared a disulfide-containing curing agent via the condensation of -NH_2_ and methyl carboxylate groups, and the curing agent was used to crosslink with epoxy resin. Five different samples (PD-1 to -5) of the curing agent were obtained by adjusting the molar ratio of DTPA and PEA. Disulfide bonds, which bring about the self-healing property to polymer backbones, can be testified by Raman spectrum and ^1^H-NMR spectrum. The key functional groups including -CO-NH-in the obtained curing agent are also confirmed by FTIR spectra. According to the results of thermal analyses (TGA and DSC) of cured samples, it was concluded that the products have excellent thermal stability as well as a *T*_g_ value lower than room temperature, displaying a good flexibility in the structure. The self-healing process was realized at a relatively low temperature (60 °C), which provided a good self-healing efficiency up to 98%. This result suggests the prepared resin samples can maintain a satisfied mechanical property after multiple repairments. The coating samples fabricated by epoxy and the disulfide-containing curing agent also demonstrated good self-healing performance, especially when the molar amount of PEA is twice that of DTPA. It is not difficult to notice that the best self-healing performance is mainly controlled by the equilibrium of dynamic bonds (-S-S-) and flexible molecular structure (polyether). Therefore, we proposed a novel route to synthesize self-healing epoxy polymer for coating applications. Rebuilding the compromise of dynamic bonds and flexible segments is expected to fabricate products showing more satisfactory self-healing performance.

## Figures and Tables

**Table 1:** Formulations of disulfide-containing curing agents (molar ratio).

Sample	PD-1	PD-2	PD-3	PD-4	PD-5
PEA: DTPA	1.8	1.9	2	2.1	2.2

**Table 2:** Coating properties of cured disulfide-containing epoxy resin.

Curing Agent	Mechanical Properties		Resistance to Liquid Medium (72 h)					
	Adhesion(N/A)	Pencil Hardness(N/A)	H_2_O	H_2_SO_4_	NaOH	NaCl	EtOH	THF
PD-1	0	2H	△	▲	△	△	▲	▲
PD-2	0	HB	△	▲	△	△	▲	▲
PD-3	0	H	△	▲	△	△	○	▲
PD-4	0	B	△	▲	△	△	▲	▲
PD-5	0	B	△	▲	△	△	▲	▲

△—sample is good without color change, swelling and breakage; ▲—sample shows significant color change, swelling and breakage; ○—sample shows slight change, as mentioned above. The used concentration of liquid medium is 1 mol·L ^−1^.

## Data Availability

Not applicable.
